# Scale-Free Dynamics in Animal Groups and Brain Networks

**DOI:** 10.3389/fnsys.2020.591210

**Published:** 2021-01-20

**Authors:** Tiago L. Ribeiro, Dante R. Chialvo, Dietmar Plenz

**Affiliations:** ^1^Section on Critical Brain Dynamics, National Institute of Mental Health, National Institutes of Health, Bethesda, MD, United States; ^2^Center for Complex Systems and Brain Sciences (CEMSC3), Instituto de Ciencias Físicas, (ICIFI) Escuela de Ciencia y Tecnología, Universidad Nacional de San Martín (UNSAM), Buenos Aires, Argentina; ^3^Consejo Nacional de Investigaciones Científicas y Técnicas (CONICET), Buenos Aires, Argentina

**Keywords:** correlations, criticality, brain dynamics, neuronal network, flocking, scale-free, synchronization, mutual information

## Abstract

Collective phenomena fascinate by the emergence of order in systems composed of a myriad of small entities. They are ubiquitous in nature and can be found over a vast range of scales in physical and biological systems. Their key feature is the seemingly effortless emergence of adaptive collective behavior that cannot be trivially explained by the properties of the system's individual components. This perspective focuses on recent insights into the similarities of correlations for two apparently disparate phenomena: flocking in animal groups and neuronal ensemble activity in the brain. We first will summarize findings on the spontaneous organization in bird flocks and macro-scale human brain activity utilizing correlation functions and insights from critical dynamics. We then will discuss recent experimental findings that apply these approaches to the collective response of neurons to visual and motor processing, i.e., to local perturbations of neuronal networks at the meso- and microscale. We show how scale-free correlation functions capture the collective organization of neuronal avalanches in evoked neuronal populations in nonhuman primates and between neurons during visual processing in rodents. These experimental findings suggest that the coherent collective neural activity observed at scales much larger than the length of the direct neuronal interactions is demonstrative of a phase transition and we discuss the experimental support for either discontinuous or continuous phase transitions. We conclude that at or near a phase-transition neuronal information can propagate in the brain with similar efficiency as proposed to occur in the collective adaptive response observed in some animal groups.

## Introduction

The collective movement of animal groups has been the subject of great interest for many decades, with the early work focusing on model simulations (Aoki, 1982; Reynolds, 1987). It is now well-accepted that collective properties in animal groups are closely related to the general study of collective phenomena in physics, which initially was focused on phase transitions in equilibrium systems composed of many, locally interacting particles (Stanley, 1971; Ma, 1976, 1985), but eventually was expanded to include far-from-equilibrium systems (Meakin, 1987; Kertesz and Wolf, 1989; Martys et al., 1991). Many biological systems were found to fit into this latter category specifically when considering systems of self-driven particles to model movements of ants (Millonas, 1992; Rauch et al., 1995), fish schools (Huth and Wissel, 1992) and bird flocks resulting in the seminal model by Vicsek et al. (1995) for flocking in biological systems based on local interactions impacted by noise. Since then, variations of the Vicsek model (Grégoire and Chaté, 2004; Chate et al., 2008) as well as other models that utilize attraction and distance rules (Couzin et al., 2002; Romanczuk et al., 2009) have been combined with experimental observations to capture population dynamics of many species such as locust swarms (Huepe et al., 2011), ants (Gelblum et al., 2016), fish schools (Tunstrøm et al., 2013), migrating white storks (Nagy et al., 2018), and cycling pelotons (Belden et al., 2019) with a major goal to understand the emergence of collective behavior from the mechanistic interactions between individuals [for a review, see e.g., Wang and Lu (2019)].

These observations support the idea that biological systems seem to be naturally poised near a phase transition (Bak, 1996), where they might benefit from order yet maintain adaptability to changing environmental conditions, an idea that is increasingly gaining attraction including the brain (Chialvo, 2010; Mora and Bialek, 2011; Plenz, 2012; Hesse and Gross, 2014; Plenz and Niebur, 2014). The initial theoretical debate has been enriched recently by an ever-improving ability to simultaneously track many biological elements (neurons, birds, midgets, etc.) over time, such that now the ideas are being challenged and contrasted by the experimental findings in the usual manner of statistical mechanics.

**Figure 1:**
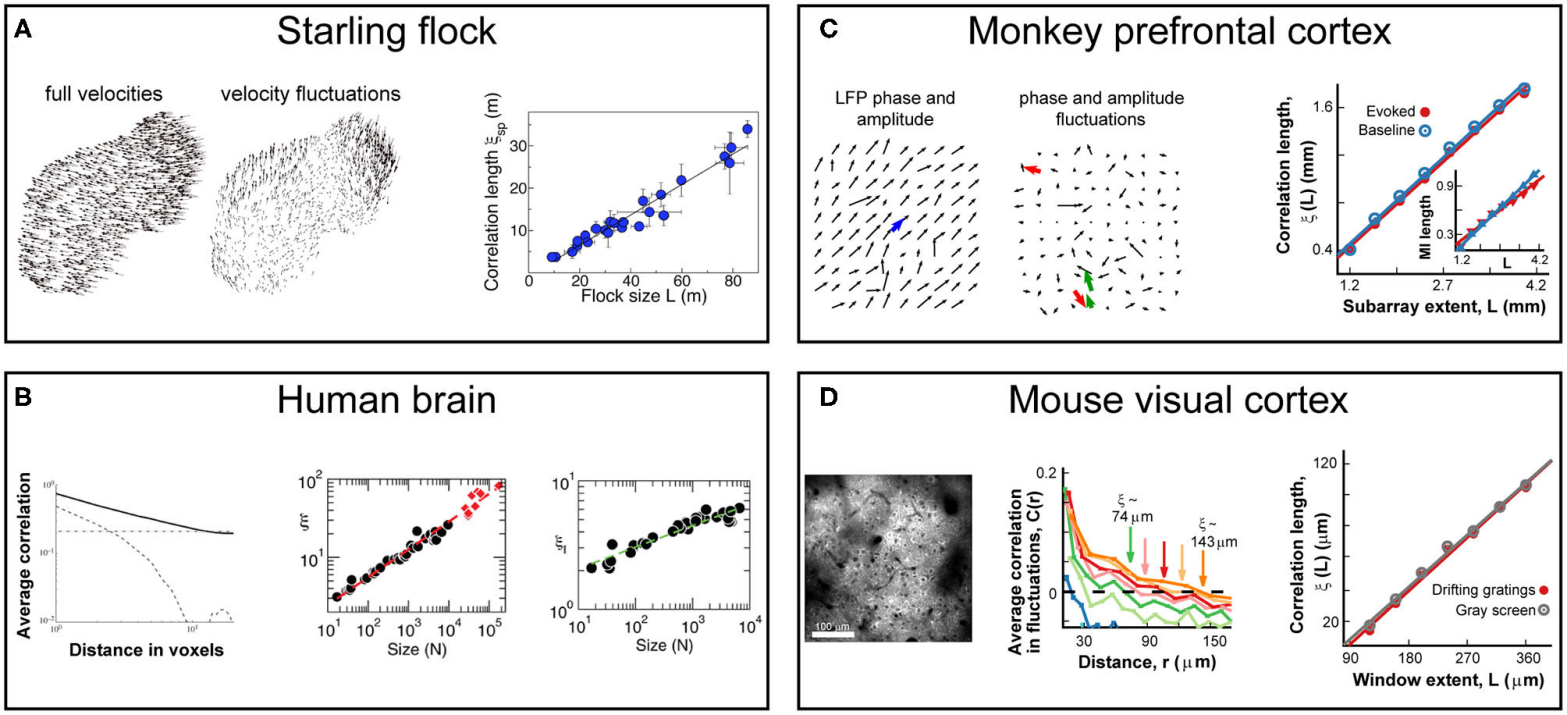
Scale-free growths in correlations length is observed in bird flocks and the mammalian brain at different scales and using different recording techniques. **(A)** Correlations in the velocity fluctuations of pairs of starlings in flocks of different sizes. Fluctuations are obtained by subtracting from each bird's velocity vector (*left*) the center-of-mass velocity of the flock (*middle*). Correlation length, defined as the distance at which correlations of the fluctuations reaches zero, scales linearly with flock size (*right*) in line with expectations from critical dynamics. Adapted from (Cavagna et al., 2010). **(B)** Correlations obtained from blood oxygenated level dependent (BOLD) signals using fMRI to measure ongoing neuronal activity of the human brain. *Left*: Average correlation between voxel pairs drops with distance between voxels as a power law (*solid line*), while phantom data drops exponentially (*dashed line*) and spatially shuffled data is constant (*dotted line*). Adapted from (Expert et al., 2011) *Middle*: Correlation length, ξ, from fluctuations in BOLD data scales linearly with the size of the brain area observed (*black circles*) or when pooling areas together (*red diamonds*). Adapted from Fraiman and Chialvo (2012). *Right*: Mutual information between voxel pairs decays with pair distance, allowing for the definition of “mutual information length,” ξ_I_, in analogy to correlation length. ξ_I_ scales linearly with the size of the brain area observed (*black circles*). Adapted from Fraiman and Chialvo (2012). **(C)** Correlations in the fluctuations of LFP amplitudes from prefrontal cortex in nonhuman primates during a working-memory task using high-density microelectrode arrays. *Left/middle*: LFP vectors depicting phase and amplitude on the array without/with subtraction of the population average (*blue arrow, left*) in analogy to velocity distributions in flock data. *Right*: Correlation length scales linearly with (sub)array size for both ongoing (*blue*) and evoked (*red*) data. Adapted from Ribeiro et al. (2020). *Inset*: Mutual information length scales linearly with (sub)array size for both ongoing (*blue*) and evoked (*red*) data. **(D)** Correlations in the fluctuations of neuronal activity from primary visual cortex in mice during visual stimulation using 2-photon imaging. *Left*: Example field-of-view showing cells used for the analysis. *Middle*: Average correlation of activity fluctuations between pairs of neurons decays with distance as well as with the size of the observed window (*colors*). *Right*: Correlation length scales linearly with observed window size for both gray screen (*gray*) or drifting gradings (*red*). Adapted from Ribeiro et al. (2020).

In this note, we focus on the behavior of the system correlation properties, the central tenet of statistical mechanics. For the sake of discussion, our starting point will be the work by Cavagna et al. in 2010, who demonstrated that starlings in a flock exhibit spatial correlations much longer than the length of direct interactions between neighboring birds (Cavagna et al., 2010, 2018). Specifically, they showed that the correlation length, i.e., the distance at which correlations drop below zero, grows monotonically with flock size (Figure 1A) and is, therefore, scale-free. The absence of any characteristic scale in the correlations is known to be a hallmark of critical systems (Wilson, 1979). For the human brain, early evidence of scale-free correlation functions was found for ongoing neuronal activity assessed indirectly using the blood oxygen level dependent signal (BOLD) (Expert et al., 2011) followed by the demonstration of correlation length to grow with the size of the observed brain region (Figure 1B) (Fraiman and Chialvo, 2012), exactly as was described for starling flocks. These remarkable population-spanning correlations were replicated for a network model of the brain with experimentally based interareal connectivity when the network dynamics was tuned to criticality (Haimovici et al., 2013). Since then, they have also been observed for bacterial colonies (Chen et al., 2012), insect swarms (Attanasi et al., 2014b), and globular proteins (Tang et al., 2017, 2020). Here, we explore specifically the analogy in scale-free correlations between animal groups and brain dynamics at the scale of local population activity during motor outputs in nonhuman primates and down to the cellular scale of single neuron interactions during sensory processing in mice. We will demonstrate that this analogy goes beyond phenomenology and shares the same formal scaling relations which suggest common underlying principles.

## Scale-Free Correlations in Response to External Perturbations

The absence of a central control for the emergence of order lies at the heart of collective phenomena. With respect to animal groups this remarkable feature is also known as “coordination” and allows animals to stay together for protection in the face of predators (Powell, 1985; Terborgh, 1990; Krause and Ruxton, 2002) or to enhance foraging (Krebs, 1973; Munn and Terborgh, 1979; Greenberg, 2000). This collective response thus requires information about a local predator or local food source to be translated into a coordinated flock response for escape behavior or foraging to be successful. Several studies have now demonstrated how swarms can achieve such de-centralized coordination using local interactions between neighbors (Gregoire et al., 2003; Sumpter, 2006; Strombom, 2011; Bialek et al., 2012; Vicsek and Zafeiris, 2012; Ling et al., 2019b).

Predominantly local interactions are also characteristic for many brain networks, specifically as found for the cortex in mammals (Markram et al., 2015). Like a bird in a flock, the “action” or output of a cortical neuron depends largely on the activity of its intracortical neighbors (Boucsein et al., 2011). The response to external perturbations of a flock, e.g., by the local intrusion of a predator, also invite interpretations similar to the response of a cortical network to external inputs. Those inputs directly affect only a small proportion of all neurons, e.g., through input from the thalamus (Bruno and Sakmann, 2006) or from other cortical regions, and thus are analogous to local perturbations of ongoing network dynamics (Arieli et al., 1996). And although neurons in a network do not change physical positions in relation to one another like birds, they may change their interaction neighborhood over time by strengthening or weakening their direct connections through synaptic plasticity. The mechanisms by which neuronal networks can propagate information quickly and flexibly to very distant, but not directly interacting, neurons are less clear though. Thus, inspired by the flock results we searched for evidence of scale-invariant correlations in brain activity in response to sensory input.

We recently explored the behavior of neuronal correlation functions at scales closer to direct neuronal interactions (Ribeiro et al., 2020). At the scale of a cortical area (i.e., the mesoscale of millimeters), we measured the distribution of the so-called local field potential (LFP) with high-density microelectrode arrays implanted in the premotor and prefrontal cortices of non-human primates performing a self-initiated motor task and a working memory task, respectively. The LFP extracts the local synchronization of neuronal groups and its emergence and propagation thus tracks the spatiotemporal evolution of population activity at a spatial resolution of several 100 μm with millisecond precision. At the scale of the cortical microcircuit (i.e., the scale of few micrometers), we measured the intracellular calcium dynamics in pyramidal cells expressing the genetically encoded calcium indicator YC2.6 in superficial layers of the primary visual cortex in awake mice passively viewing drifting gratings. The fluorescent indicator closely tracks the action potential firing in individual pyramidal neurons, which allows for a cellular reconstruction of spatiotemporal population activity with micrometer spatial resolution and sub-second temporal precision. At both scales, we observed the linear growth of the correlation length as a function of the linear size of the sampled area during sensory processing and motor output (Figures 1C,D). Remarkably, these scale-free correlations were similarly present during rest and evoked responses from the sensory/motor stimulation (Figures 1C,D) [see also Ribeiro et al. (2020)]. In line with previous results for the whole brain (Fraiman and Chialvo, 2012), the mutual information found in neuronal activity also behaved in a scale-free manner. By measuring how the mutual information between pairs of electrodes decays with distance, we showed that the “mutual information length” grew linearly with system size, just like the correlation length, for ongoing and evoked neuronal at the mesoscale (Figure 1C, inset).

Animal groups exhibit collective behavior in space during motion, in contrast to the brain, where activity propagates in high-dimensional networks and neurons themselves are stationary. These differences come into focus when considering scaling of correlation length by the spontaneous breaking of continuous rotational symmetry as is the case for orientation in space. In this case, global ordering can emerge in the absence of criticality at lower temperatures including the presence of powerlaw decay in space (Goldstone's theorem) (Goldstone, 1961). For this reason, Cavagna et al. (2010) also investigated correlations in the speed of birds, for which that argument does not apply: whereas orientation could be seen as a soft mode (being bound, they have a “soft” degree of freedom), speed in principle is unbounded and thus is considered a so-called “stiff” mode. In the case of brain activity, the Goldstone's theorem does not apply, at least for the data presented here, since there is no continuous symmetry that can be broken or soft modes. It needs to be noted that although a “pseudo” phase can be extracted from the LFP using a Hilbert transform of the original time series (Yu et al., 2017) the work of Ribeiro et al. (2020) used only the change in LFP amplitude (which is unbounded) to compute the correlation length. Furthermore, similar results were obtained when using binarized negative excursions of the LFP below a certain threshold [so-called nLFPs, which represent the local, synchronous firing of neurons around the electrode; see Yu et al. (2017)], calcium traces or deconvolved spikes (Ribeiro et al., 2020), all of which are analogous to speed in animal movement.

## Interaction Length vs. Correlation Length

As commented, many animals living in groups synchronize their behavior to that of their neighbors. In that manner, they can spend less time on the lookout for predators and more time feeding or resting (Bednekoff and Lima, 1998). If animals were required to be on alert to the behavior of distant group members, more resources would need to be allocated to group observation. Obviously, this requirement might not even be possible, e.g., for herds that are confined to a plane where observation of distant members is obscured or for very large animal groups in general. The attention toward neighbors is accounted for by most models of collective behavior in animal groups, which, considering local interactions (Vicsek et al., 1995; Cucker and Smale, 2007; Wang and Lu, 2019), are able to capture the synchronization of animals to their neighbors as found for red deer (Rands et al., 2014) and recently for black-headed gulls (Evans et al., 2018). In a more extreme example, mosquitofish were shown to only respond to their single nearest neighbor (Herbert-Read et al., 2011). Thus, regardless of whether interactions between animals depend on metric or topological distance (Ballerini et al., 2008; Ginelli and Chate, 2010; Strandburg-Peshkin et al., 2013), it is probably safe to say that keeping track of nearby neighbors is a preferred behavioral strategy in groups. On the other hand, this strategy requires that information pertinent for the individual survival must travel efficiently throughout the entire group, independently of the group size. In physics, this feature of transforming local (short-range) interactions into global (long-range) correlations, is known to be present in systems (almost exclusively) at criticality (Wilson, 1979). Support for this concept comes from the work of Cavagna et al. (2015) who employed a maximum entropy approach to infer the effective interactions from individuals in a natural flock and showed that the interaction range decays exponentially over the range of just a few individuals. Additionally, Calvão and Brigatti's model (Calvao and Brigatti, 2019), which is an implementation of the classical “selfish herd hypothesis” (Hamilton, 1971), is composed of local-interacting agents which collectively undergo a discontinuous phase transition. Their model successfully reproduces the behavior observed in nature for midge swarms including long-range correlations (Attanasi et al., 2014a,b).

For the brain, direct interactions between neurons exhibit a far more complex and selective organization than nearest neighbor relations. Neuronal interaction in the cortex includes a dominant number of direct short-range connections onto which long-range connections are superimposed that link distant cortical regions within and between hemispheres. Accordingly, the observation of long-range correlations might arise from short-range interactions at critical dynamics, from long-range connections independent of dynamical regimes, or both. To disambiguate this, we have simulated critical dynamics in a neuronal network with a precisely defined characteristic size for its connections and evaluated how the correlation function changes for distances beyond the short interaction range (Ribeiro et al., 2020). We found that there is a clear change in the behavior of the correlation function at the interaction range, with correlations growing much faster for distances up to this point, confirming our experimental findings in primary visual cortex. The obtained interaction distance was similar to the characteristic distance at which two pyramidal cells in layers II/III are connected anatomically (Levy and Reyes, 2012; Seeman et al., 2018). These results suggest critical dynamics in combination with short interactions to be a major factor behind the observed correlation length scaling at the microscale and indirectly suggest that as in the case of animal flocks, the information about a local input or perturbation can rapidly propagate through the entire system.

## Effects of the Heterogeneity of the Elements on the Correlation Structure

Although some early works have taken heterogeneity and self-sorting into account (Couzin et al., 2002), animal group behavior has been mostly studied assuming homogeneous behavior of the individual (Ero et al., 2018; Gouwens et al., 2019). More recently, the effects of heterogeneity within groups has gained increased attention [for a review, see e.g., King et al. (2018)]. For instance, it has been shown that body size affects the strength of social interactions and the spatial organization of fish schools (Romenskyy et al., 2017). For jackdaws, a bird species that form lifelong pair-bonds, social relationships between different birds lead to the appearance of sub-structures within a flock. Pair-bonded jackdaws interact with fewer neighbors than unpaired birds, flap their wings more slowly, which may save energy and flocks with more pairs exhibit shorter correlation length, which may lead to decreased group-level benefits (Ling et al., 2019a).

For the mammalian brain, already a cortical column with ~10,000 neurons across its six layers provides a major modeling challenge with its diversity in cell types, cell connectivity, cellular, and subcellular dynamics (Markram et al., 2015; Dura-Bernal et al., 2019). The type of dynamics that in principle can be generated in these high-dimensional models is not easily constrained and can range from large-scale synchronized oscillations to more local, sometimes sequential activity. With respect to the latter and in analogy to how the social relationships affect correlations in jackdaw flocks (Ling et al., 2019a), it has been shown that some neurons (leaders) consistently fire earlier than others in spontaneous bursts of activity *in vitro* (Eytan and Marom, 2006; Eckmann et al., 2008; Orlandi et al., 2013; Pasquale et al., 2017). Yet, it is currently not known how the heterogeneity of cell types, layers and areas contribute to scale-free correlation lengths measured in the awake brain at macro-, meso-, and microscale. In a first attempt to address this issue, we studied functional subnetworks in cortical circuits, such as the one formed by orientation selective, i.e., “tuned” cells with similar tuning preference in V1 (Palagina et al., 2019). When separately analyzing tuned and non-tuned cells, despite significant changes in the absolute value of correlation changes (evidencing the different structure present in these subgroups), we were able to show that scale-free correlations are present along the tuning dimension (Ribeiro et al., 2020). We note that the subgroup not included in the analysis was still participating in the overall network response and that this finding does not exclude the possibility that both subgroups are essential to create the observed scale-free correlations for both subnetworks.

## Nature of the Phase Transition Underlying the Collective Properties of Animal Groups and Neuronal Populations

A variety of collective states can be observed in animal groups. For instance, Tunstrøm et al. (2013) have shown that golden shiner schools can present three dynamically-stable collective states, namely swarm, polarized and milling, with frequent transitions between them. Naturally, different types of collective states and accompanied transitions between those states are necessary for different animal species. Here, we discussed coordination or synchronization in animal groups in the context of emerging of directional order (or onset of collective motion) (Vicsek and Zafeiris, 2012) and condensation or clustering transitions (Chen et al., 2012; Calvao and Brigatti, 2019). Modeling work reflects this wide variety in collective behavior, which have been linked to different types of phase transitions, mainly of the discontinuous type [first order transitions, including hysteresis and metastability; e.g., Couzin et al. (2002), Chate et al. (2008), Hein et al. (2015), and Calvao and Brigatti (2019)] or the continuous type [second order, in line with criticality; e.g., Barberis and Albano (2014), Calovi et al. (2015), Feinerman et al. (2018)], or both (Huepe et al., 2011). Even within one model, the type of phase transition encountered can be sensitive to the specific model parameters and simulations conducted. For example, the original introduction of the Vicsek model (Vicsek et al., 1995) suggested a second-order phase transition, yet, clear discontinuities where identified particularly when adding aggregational terms and/or allowing noise to be directly added to the neighborhood computation (Grégoire and Chaté, 2004; Chate et al., 2008). There is strong theoretical evidence for the discontinuous nature of the transition in the Vicsek model (Bertin et al., 2009; Ihle, 2011; Peshkov et al., 2014), yet finite-size effects can smooth a discontinuous transition making it appear continuous (Grégoire and Chaté, 2004; Solon et al., 2015; Brown et al., 2020). Nevertheless, there are claims for the existence of robust continuous transitions in the Vicsek model within certain parameter regimes (Barberis and Albano, 2014). These commonly encountered sensitivities of abstract models to parameter regime and seemingly innocent model variation, necessarily call for elaborate experimental designs to validate models. For example, cooperative transport in ants was found to be more in line with a continuous phase transition when quantifying transport velocity for food pellets of different sizes (Feinerman et al., 2018).

The plethora of models that can be construed for brain networks ranging from abstract, binary neurons with random connectivity to detailed compartmental neuronal networks requires a prudent and stepwise alignment of theory and models with continuously improving experimental evidence. Here, we would like to point out the experimental demonstration of scale-free neuronal avalanches in isolated brain preparations in line with predictions for a critical branching process (Beggs and Plenz, 2003). This experimental finding suggested that system wide correlations form spontaneously in a fluctuation dominated brain state, with low and sparse rate. The experimental demonstration of scale-free (most-often weak) correlations for spontaneous and evoked neuronal activity in the awake brain in the presence of scale-invariant neuronal avalanches has been reliably found at the macroscale (Expert et al., 2011; Fraiman and Chialvo, 2012; Tagliazucchi et al., 2012), meso and microscale (Ribeiro et al., 2020). Importantly, LFP avalanches in the non-human primate that show scale-free correlations also exhibit a scaling collapse with an exponent of 2 for mean size vs. duration and an inverted parabolic profile in line with predictions for a critical branching process (Miller et al., 2019). This scaling collapse revealed a complex interaction with simultaneously present oscillations exhibiting the value of 2 at temporal resolutions outside the scale of the oscillation, required limit-analysis when measured at the scale of the oscillation, and collapsed to 1.5 when oscillations were removed by low-pass filtering. It is this body of experimental results in the awake cortex (Scott et al., 2014; Bellay et al., 2015), which forms the seed for a more comprehensive understanding of the mechanisms ruling the scale-free dynamics in brain activity.

A variety of alternative models and simulations often exhibit significant differences when accounting for the above-mentioned body of experimental findings. For example, the identification of universality classes that deviate from the directed percolation model have been found to be indecisive to explain neural data obtained from the anesthetized or sleep state under severe subsampling conditions (Fontenele et al., 2019; Carvalho et al., in press). Similarly, neuronal models that feature a first order transition between a low and high activity mode switched randomly by external noise and include oscillations (Scarpetta and de Candia, 2013; Scarpetta et al., 2018), while demonstrating a size distribution exponent of −3/2, also exhibit scaling exponents ~1.1, which is lower than the relationship found in awake nonhuman primates (Miller et al., 2019). The Landau-Ginzburg scenario introduced recently to simulate avalanches in neuronal networks (di Santo et al., 2016; Buendia et al., 2020) exhibits, under certain parameter choices, a first order transition, hysteresis, and exponents similar to those of a critical branching process. However, the temporal avalanche profile identified in that model differs from an inverted-parabola measured experimentally in non-human primates (Miller et al., 2019). In addition, the disorder-synchronization phase transition in that model gives rise to statistically distinct giant (“king”) avalanches found typically in disinhibited brain activity similar to epileptic seizures.

As a final reflection on this aspect, it needs to be noted that in contrast with the empirical solitude of the finding of neuronal avalanches a decade and half ago, the field is currently populated by a large variety of not-always self-consistent models. It seems that a fruitful avenue now might be to balance the modeling efforts with a careful analysis of the continuously improving sophisticated experimental evidence at hand.

## Importance of Scale-Free Correlations for Brain Function

**Figure 2:**
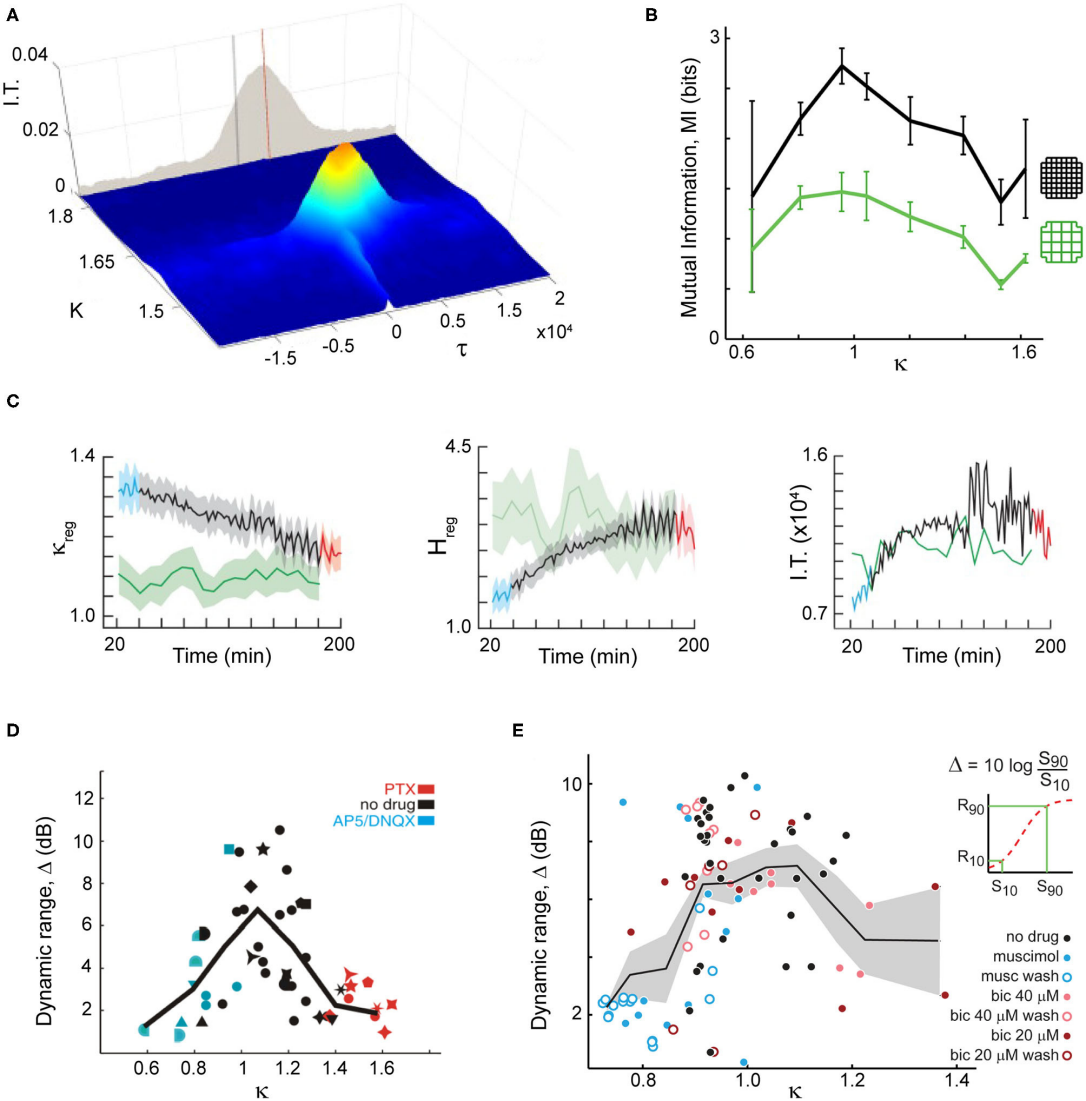
Criticality and scale-free organization provide key advantages for both flocks and brains such as maximal information transmission and dynamic range. **(A)** In a decision-making model of flock behavior, information transmission peaks at criticality, shown by a peak in mutual information for *K* ~ 1.62 and τ ~ 2,500 (critical point). Adapted from Lukovic et al. (2014). **(B)** In organotypic cultures grown from rodent brains, information transmission peaks when spontaneous neuronal activity displays scale-free neuronal avalanches, in line with expectation from criticality (κ ~ 1). Two different coarse-graining levels are shown (*colors*). Proximity to criticality, i.e., scale-free avalanches, is controlled through pharmacological manipulation of the cultures. Adapted from Shew et al. (2011). **(C)** Information transmission is maximized as mice recover from anesthesia, establishing neuronal avalanches. *Left*: Criticality distance measure approaches 1 (critical point) as time from anesthesia application (in min) passes. Anesthetized (*blue*), recently awake (*red*) and fully awake (*green*) states are highlighted. Entropy (*middle*) and information transmission (*right*) reaches a maximum as mice recover from anesthesia and reestablishing neuronal avalanches. Adapted from Fagerholm et al. (2016). **(D)** In organotypic cortex cultures, the dynamic range peaks when neuronal avalanches emerge and can be reduced when pharmacologically changing the natural excitation/inhibition balance. Adapted from Shew et al. (2009). **(E)** Using microelectrode array recordings in rats *in vivo*, the peak of dynamic range was demonstrated using natural stimuli and changes in excitation/inhibition balance through local pharmacological manipulation. Adapted from Gautam et al. (2015).

A large body of modeling work and some experimental evidence have shown that scale-free correlations are beneficial, providing key advantages to animals living in groups. For example, Rauch et al. (1995) showed the emergence of self-organized trails near a critical density of foraging ants. The length of these trails exceeded several orders of magnitude the ants perceptual scale, being another example of long-range correlations. In the same line, it has been shown that evolutionary pressure could move fish schools toward an optimized state near a discontinuous phase transition in an evolutionary model, where local environmental perturbations can cause changes in the collective school state (Hein et al., 2015). Using the Vicsek model for flocks (Vicsek et al., 1995), it has been shown that information transmission is maximized near the phase transition (Figure 2A) (Vanni et al., 2011; Lukovic et al., 2014), which as discussed in the previous subsection could have an underlying first-order origin. The enlarged correlations arising as a result from this maximized information transmission, lead to optimized response to predators (Mateo et al., 2017), in line with what has been observed in data-driven models of fish schools (Calovi et al., 2015) or sheep herds (Ginelli et al., 2015) near criticality. It has also been shown that the efficiency of computations in the Grégoire and Chaté (2004) model is maximized at the phase transition (Crosato et al., 2018).

On the brain side, theory and model simulations on critical dynamics in neuronal networks has proposed many advantages in information processing, some of which have been demonstrated experimentally, specifically when using pharmacological manipulations to move cortical networks away from neuronal avalanche dynamics (Figures 2B–E) [for reviews, see e.g., Shew and Plenz (2013) and Cocchi et al. (2017)]. For example, the dynamic range, which measures the range of stimulus intensity a network is able to differentiate, has been proposed to maximize at criticality by Kinouchi and Copelli (2006) and was demonstrated experimentally (Figures 2D,E) (Shew et al., 2009; Gautam et al., 2015).

Another parallel between scale-free flocks and brains is the presence of decentralized signal processing. This aspect has gained increased attention in the context of artificial intelligence, with many studies proposing the usage of artificial swarm systems (Hornischer et al., 2019; Sueoka et al., 2019). The brain also provides inspiration for these systems: Monaco et al. (2020) proposed an analogy between these multi-agent robotic platforms and place cells in the hippocampus, suggesting improvements to current models that follow solutions found by brain circuits. Startle responses in animal populations can trigger escape waves (Herbert-Read et al., 2015; Sosna et al., 2019), in the latter case yielding heavy-tail cascade size distributions and involve distributed repositioning of in the swarm beyond an individual's sensitivity changes to perturbation. The initiation and spread of such local response bears similarities to branching process dynamics suggesting promising similarities with critical brain dynamics.

## Conclusions

The emergence of order in systems composed of a myriad of small entities exhibits many parallels between animal groups and neuronal populations in the brain. We summarized new experimental findings for the brain on the emergence of scale-invariant correlations and scale-invariant population sizes and discussed their similarities and differences compared to collective behavior in animals. We show that for both fields of research there are fascinating arguments for systems to be positioned near a phase transition to support propagation of local information throughout the entire system. Future experimental work on the role of cell types and microcircuit mechanisms in maintaining these scale-free dynamical features are crucial for understanding how the brain processes information.

## Data Availability Statement

The raw data supporting the conclusions of this article will be made available by the authors, without undue reservation.

## Ethics Statement

The animal study was reviewed and approved by the NIMH Animal Care and Use Committee.

## Author Contributions

All authors have made a substantial, direct and intellectual contribution to the work, and approved it for publication.

## Conflict of Interest

The authors declare that the research was conducted in the absence of any commercial or financial relationships that could be construed as a potential conflict of interest.
